# Safety and Tolerability of Oral Cannabinoids in People Living with HIV on Long-Term ART: A Randomized, Open-Label, Interventional Pilot Clinical Trial (CTNPT 028) †

**DOI:** 10.3390/biomedicines10123168

**Published:** 2022-12-07

**Authors:** Ralph-Sydney Mboumba Bouassa, Judy Needham, Dana Nohynek, Joel Singer, Terry Lee, Florian Bobeuf, Suzanne Samarani, Lina Del Balso, Natalie Paisible, Claude Vertzagias, Giada Sebastiani, Shari Margolese, Enrico Mandarino, Marina Klein, Bertrand Lebouché, Joseph Cox, Marie-Josée Brouillette, Jean-Pierre Routy, Jason Szabo, Réjean Thomas, Emmanuel Huchet, Antonio Vigano, Mohammad-Ali Jenabian, Cecilia T Costiniuk

**Affiliations:** 1 Department of Biological Sciences and CERMO-FC Research Centre, Université du Québec à Montréal, Montreal, QC H2X 3Y7, Canada; 2 Infectious Diseases and Immunity in Global Health Program, Research Institute of McGill University Health Centre, Montreal, QC H4A 3J1, Canada; 3 CIHR Canadian HIV Trials Network, Vancouver, BC V6Z 1Y6, Canada; 4 Centre for Health Evaluation and Outcome Sciences, St. Paul’s Hospital, Vancouver, BC V6Z 1Y6, Canada; 5 School of Population and Public Health, University of British Columbia, Vancouver, BC V6T 1Z4, Canada; 6 Department of Medicine, Division of Infectious Diseases and Chronic Viral Illnesses Service, McGill University Health Centre, Montreal, QC H4A 3J1, Canada; 7 Department of Medicine, Division of Gastroenterology and Hepatology, McGill University Health Centre, Montreal, QC H4A 3J1, Canada; 8 Department of Family Medicine, McGill University Health Centre, Montreal, QC H4A 3J1, Canada; 9 Canadian Institutes of Health Research Strategy for Patient-Oriented Research Mentorship Chair in Innovative Clinical Trials, Montreal, QC H4A 3J1, Canada; 10 Department of Psychiatry, McGill University Health Centre, Montreal, QC H4A 3J1, Canada; 11 Department of Medicine, Division of Hematology, McGill University Health Centre, Montreal, QC H4A 3J1, Canada; 12 Clinique Médical L’Actuel, Montreal, QC H2L 4P9, Canada; 13 Clinique Médical L’Agora, Montreal, QC H2L 4E9, Canada; 14 Medical Cannabis Program in Oncology, Cedars Cancer Center, McGill University Health Centre, 1001 Boulevard Decarie, Montreal, QC H4A 3J1, Canada; 15 Centre for Cannabis Research, McGill University, Montreal, QC H3A 0G4, Canada; 16 Department of Microbiology, Infectiology and Immunology, Université de Montréal, Montreal, QC H3T 1J4, Canada; 17 Department of Microbiology and Immunology, McGill University, Montreal, QC H3A 0G4, Canada

**Keywords:** HIV, cannabinoids, cannabidiol (CBD), tetrahydrocannabinol (THC), chronic liver diseases, quality of life, pilot clinical trial

## Abstract

Background: With anti-inflammatory properties, cannabinoids may be a potential strategy to reduce immune activation in people living with HIV (PLWH) but more information on their safety and tolerability is needed. Methods: We conducted an open-label interventional pilot study at the McGill University Health Centre in Montreal, Canada. PLWH were randomized to oral Δ9-tetrahydrocannabinol (THC): cannabidiol (CBD) combination (THC 2.5 mg/CBD 2.5 mg) or CBD-only capsules (CBD 200 mg). Individuals titrated doses as tolerated to a maximum daily dose THC 15 mg/CBD 15 mg or 800 mg CBD, respectively, for 12 weeks. The primary outcome was the percentage of participants without any significant toxicity based on the WHO toxicity scale (Grades 0–2 scores). Results: Out of ten individuals, eight completed the study. Two from the CBD-only arm were withdrawn for safety concerns: phlebotomy aggravating pre-existing anemia and severe hepatitis on 800 mg CBD with newly discovered pancreatic adenocarcinoma, respectively. Seven did not have any significant toxicity. Cannabinoids did not alter hematology/biochemistry profiles. CD4 count, CD4/CD8 ratio, and HIV suppression remained stable. Most adverse effects were mild-moderate. Conclusions: In PLWH, cannabinoids seem generally safe and well-tolerated, though larger studies are needed. Screening for occult liver pathology should be performed and hepatic enzymes monitored, especially with high CBD doses.

## 1. Introduction

People living with HIV (PLWH) experience persistent immune activation and systemic inflammation [1,2,3,4]. These processes, in turn, drive development and progression of non-Acquired Immune Deficiency Syndrome (AIDS)-related comorbidities such as cardiovascular diseases, metabolic and neurological disorders, malignancies, and liver fibrosis [1,2,3,4]. By attenuating chronic inflammation, one may slow the progression of chronic diseases [5,6,7,8].

Historically, cannabis was used by PLWH to alleviate AIDS-related symptoms such as nausea, anorexia and depression [9]. During the modern antiretroviral treatment (ART) era, cannabis use remains common amongst PLWH for both recreational and medicinal reasons, including chronic pain, anxiety and depression [9,10,11,12,13]. The primary phytocannabinoids, delta-9-tetrahydrocannabinol (THC) and cannabidiol (CBD), possess anti-inflammatory and anti-fibrotic properties, as demonstrated in vitro [14,15,16,17,18] and in vivo during animal [19,20,21,22] and human observational studies [23,24,25]. Therefore, cannabinoids might be a potential therapeutic strategy to reduce chronic inflammation in PLWH on ART.

Due to successful advocacy during the early days of the HIV/AIDS epidemic, medical cannabis first became legalized in Canada in 2001 [26]. However, cannabinoid-based medicines (CBM) have not gone through the traditional drug development and formal drug approval process, and formal nonclinical pharmacokinetic and toxicology information are lacking [7]. Consequently, there remains an important lack of safety data for cannabis-based medicines in PLWH in the modern ART era [7]. Moreover, with legalization of recreational cannabis in Canada [27], it is now easier to obtain cannabinoids which may not have been thoroughly evaluated for safety and tolerability [28,29]. Before conducting large scale clinical trials to evaluate the efficacy of CBM for diverse comorbidities and symptomatology experienced by PLWH, a pivotal step will be to determine the safety, tolerability and feasibility, of using oral cannabinoids in this population. Here, we report on the safety and tolerability of oral cannabinoids in PLWH in a randomized, open-label, interventional pilot study. A THC:CBD combination arm was selected as both compounds have therapeutic properties and may function synergistically [30,31,32,33,34]. Furthermore, the use of CBD tends to improve THC tolerability when combined [30,31,32,33,34]. A CBD-only arm was also selected, in part, to observe the effects of CBD monotherapy in addition to feasibility reasons (i.e., capsules availability). Feasibility and effects on immune cell profiles, inflammatory markers, HIV reservoir size and gut microbiome will be reported in separate manuscripts.

## 2. Methods

### 2.1. Study Design

This was a randomized, open-label, interventional pilot study (CIHR Canadian HIV Trials Network (CTN) PT028) to assess the safety and tolerability of oral THC:CBD combined or CBD-only capsules consumed daily for 12 weeks [7].

### 2.2. Enrolment, Selection Criteria and Study Population

Recruitment occurred at the Chronic Viral Illness Service, Royal Victoria Hospital of the McGill University Health Centre in Montreal, Canada. Participants were included if they were 18 years of age or older and had HIV infection with suppressed viral load (VL) <40 copies/mL on ART for at least 3 years. Participants also had to have a negative baseline cannabinoid urine screen. Participants were excluded if they used cannabinoid-containing products outside of the study or within 4 weeks of study commencement. A full list of inclusion/exclusion criteria is included in Supplementary Table S1.

### 2.3. Study Intervention

Oral capsules, manufactured by Tilray Brands, Inc. (New York City, NY, USA), consisted of highly purified (>98%) cannabinoids in oil. Formulations included TN-TC11M2, a THC:CBD combination in a 1:1 ratio (2.5 mg/2.5 mg), and TN-C200M2, consisting CBD only (200 mg). Capsules were of interest given the potential of orally administered cannabinoids to reduce gut-associated inflammation [35]. Due to person-to-person variability in metabolism and tolerability [36], participants up-titrated cannabinoid doses as tolerated (Table 1), a method which has proven successful in other clinical trials [37]. Dosage ranges for both the THC/CBD combination (2.5 to 15 mg/day) and the CBD-only formulation (200 to 800 mg/day) have been determined based on other clinical trials demonstrating safety, tolerability, and the efficacy of these doses for the management of other pathologies such as chronic pain, epilepsy, schizophrenia, or even multiple sclerosis [38,39,40,41].

### 2.4. Randomization

Participants were randomized in a 1:1 ratio to either TN-TC11M2 (arm 1) or TN-C200M2 (arm 2).

### 2.5. Safety and Tolerability Assessments and Specimen Collection

**Figure 1:**
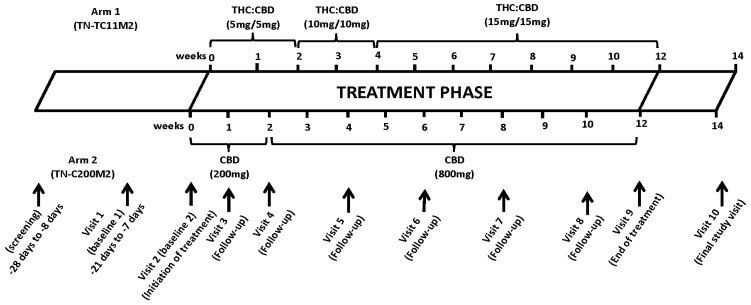
Schedule of visits and procedures. Screening: Up to 4 weeks prior to randomization, during the screening visit, study staff explained the study to the participants and obtained written informed consent prior to initiating any study procedures. Study staff assessed the participant’s eligibility by assessing the inclusion and exclusion criteria. Study staff collected the medical history and concomitant medications of the study participants and they underwent a complete physical exam. Blood was collected for hematology, blood chemistry, HIV RNA load and CD4 and CD8 T cells counts. A urine pregnancy test was performed for female participants. Cannabis Use Disorder Identification Test-Revised (CUDIT-R), Drug Use Disorder Identification Test (DUDIT) and Alcohol Use Disorder Identification Test (AUDIT) questionnaires were administrated to the participants and they underwent testing for Hepatitis B and C and syphilis infections. They also underwent urine screen for cannabinoids use. Baseline 1: Up to 3 weeks before the randomization, study staff confirmed eligibility of the candidate and reviewed their medical history. Participants then underwent a second cannabinoids screening test, if his/her initial screen was positive, and answered the CUDIT-R questionnaire in order to identify any problematic cannabis use. The participants underwent a targeted physical exam and blood and semen (from male) were collected to quantify the HIV reservoir size in circulating PBMC from blood and in the semen. Nasal swab and stool specimens were collected from study participants. Antiretroviral Therapy (ART) compliance, alcohol intake and concomitant medication were reviewed by the study staff. Baseline 2 (week 0: Initiation of treatment): Participants confirmed their willingness to participate in the study and eligibility was confirmed, before participants were randomized to either arm 1 or arm 2. Blood was collected from participants. Participants underwent a targeted physical exam. Participants also completed the World Health Organization Quality of Life—HIV Brief Scale (WHOQOLHIV-BREF), Euro-Qol-5Dimension (EQ-5D) questionnaire, and Profile of MoodStates (POMS) questionnaires before receiving a one week supply of the study medication. Follow-up visits (visit 3–8; week 1 to 10): During the follow-up visits, participants underwent a physical examination, and blood was collected to assess the biological study measures. Study drug and ART compliance was assessed. Adverse effects (AEs) were recorded. Pregnancy test was performed on urine of female participants. The participants completed the WHOQOLHIV-BREF, EQ-5D, and POMS questionnaires (Visit 6) and received the study medication until their next visit. End of the treatment (Visit 9; week 12): At Visit 9, participants underwent a physical examination, and blood was collected to assess the biological study measures. Nasal swab and stool specimens were collected from all study participants and semen was collected from male participants. AEs were recorded. A pregnancy test was performed on urine of female participants. Participants then completed the WHOQOLHIV-BREF, EQ-5D, and POMS questionnaires. Final study visit (Visit 10; week 14): At the final visit, participants underwent a physical examination, and blood was collected to assess the biological study measures. AEs were recorded and ART compliance was assessed. A pregnancy test was performed on urine of female participants.

The visit schedule is depicted in Figure 1. Participants underwent a physical exam and occurrence of adverse events (AEs), use of concomitant medications and the presence of common symptoms associated with cannabinoids (including dizziness, nausea, headaches, appetite or mood changes) were assessed. Toxicity of TN-TC11M2 and TN-C200M2 was assessed using the World Health Organization (WHO) toxicity scale. All AEs, regardless of grade, were documented, and those possibly related to TN-TC11M2 and TN-C200M2 were managed by dose reduction. Cannabinoids were permanently discontinued when life-threatening AEs occurred. Blood was drawn for CD4 and CD8 T-cells counts, plasma VL, complete blood count, aspartate aminotransferase (AST), alanine aminotransferase (ALT), alkaline phosphatase (ALP), total bilirubin, urea, creatinine and blood glucose, as well as future T-cell activation and inflammatory markers. Nasal swabs and stool specimens were collected at baseline and end of the treatment period for future microbiome analysis. Men had the option of donating a semen specimen collected at baseline and end of the treatment period for future HIV reservoir studies.

### 2.6. Quality of Life and Mood Assessment

WHO Quality of Life HIV Brief (WHOQOLHIV-BREF), Euro-Qol-5Dimension (EQ-5D) and Profile of Mood States (POMS) questionnaires were administered at baseline, midway through the study (Visit 6) and at the end of treatment (Visit 9) (Figure 1). WHOQOLHIV-BREF consists of 31 items that measure the following domains: physical health, psychological health, social relationships and environment [42]. EQ-5D is a descriptive questionnaire examining five dimensions: (1) mobility, (2) self-care, (3) usual activities, (4) pain/discomfort and (5) anxiety/depression [43]. Meanwhile, the POMS questionnaire measures the following six factors: (1) tension-anxiety, (2) anger-hostility, (3) fatigue-inertia, (4) depression-dejection, (5) vigour-activity and (6) confusion-bewilderment [44].

### 2.7. Study Outcome Measures

Endpoints consisted of (1) the proportion of participants in both groups without any sign of significant toxicity as determined by the WHO toxicity scale (i.e., number of participants with grades 0–2 scores on the WHO toxicity scale); (2) the proportions of participants who were able to complete the study and (3) changes in scores on the WHOQOLHIV-BREF scale, EQ-D5 and POMS questionnaires from week 0 to week 12. A description of WHO toxicity scale grades is presented in Supplementary Table S2.

### 2.8. Ethics

Prior to study enrolment, individuals signed a written informed consent form. This study was approved by the Research Ethics Board of the McGill University Health Centre (#2018-4336) and conducted in conformity with the Declaration of Helsinki.

### 2.9. Statistical Analyses

Data were recorded onto data collection work sheets and then entered into an InForm collection and trial management online platform. Descriptive statistics were used. Means (standard deviation) and medians (interquartile range) were calculated for quantitative variables and the non-parametric Friedman test was used to assess differences between measures repeated at each visit. Wilcoxon signed-rank test was used to compare paired repeated measurements between two visits. GraphPad Prism Software (version 9.0.0, San Diego, CA, USA) was used for statistical analyses.

## 3. Results

### 3.1. Study Participants

**Figure 2:**
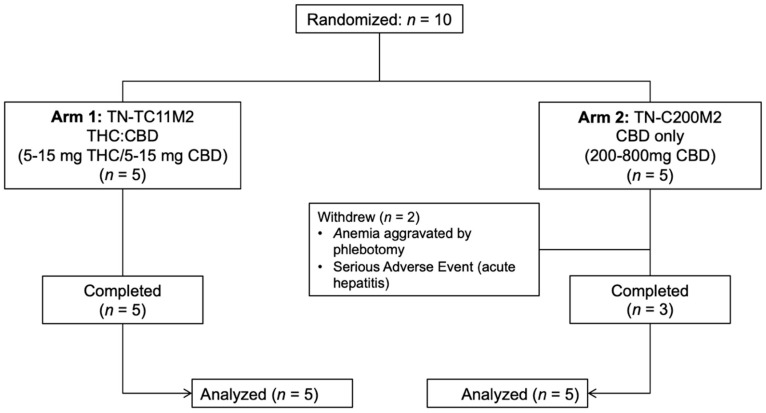
Allocation of participants enrolled in the study (*n* = 10). Distribution of study participants randomized to arm 1 (TN-TC11M2; THC:CBD) or arm 2 (TN-C200M2; CBD-only), to the study.

Between September 2021–February 2022, 10 PLWH were enrolled. The initial enrollment target was 26 participants, but the study was closed prematurely due to rupture of cannabinoid capsules stock, the impossibility of renewing the stock of capsules with the same manufacturing criteria and enrolment challenges. Baseline characteristics are summarized in Table 2. Median age was 57.5 years (IQR: 54.75–61.75) and most were male (80%). Based on the CUDIT-R results, 7 out of the 10 participants (70%) reported having consumed cannabis during the past 6 months. Study group allocation is depicted in Figure 2.

### 3.2. Safety and Tolerability

**Figure 3:**
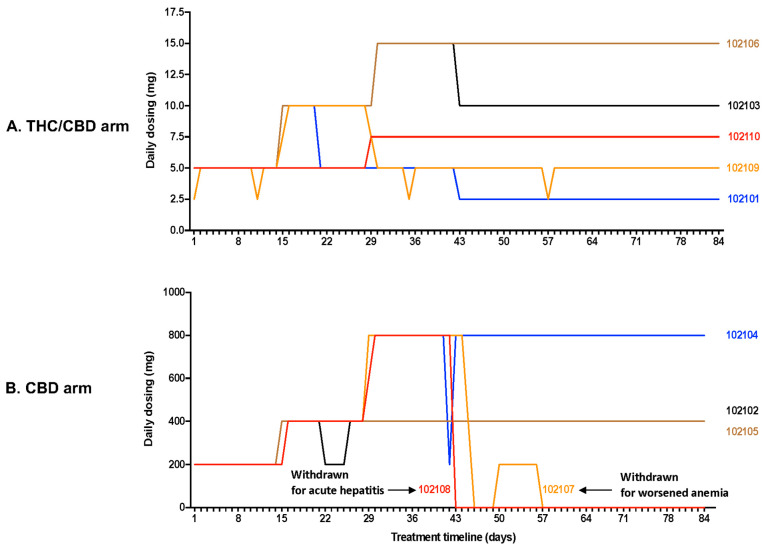
Daily dosage of CBD-only and THC/CBD combination during the 12 weeks of treatment. (**A**) THC/CBD arm: In arm 1 (THC/CBD), two participants were able to reach the maximum daily dose of the study drugs (15 mg THC/15 mg CBD), but only one remained at this dose until the end of the treatment, the other participant reduced his dosing to 10 mg THC/10 mg CBD per day because of the occurrence of AEs (somnolence). Two other participants from arm 1 reached the daily dose 10 mg THC/10 mg CBD, but after 3 weeks of treatment, they reduced their dosing because of the occurrence of AEs, one participant experienced cognitive impairment (#102109), while the other had somnolence, fatigue, difficulty concentrating, nightmares and paranoid thoughts (#102101). One remained at 5 mg THC/5 mg CBD per day and the other one who had multiple AEs reduced his daily dose to 2.5 mg THC/2.5 mg CBD after 5 weeks of treatment. A participant (#102110) from arm 1 who first reduced his daily dosing from 5 mg THC/5 mg CBD after 3 weeks of treatment to 2.5 mg THC:2.5 mg CBD, finally increased his dosing from 2.5 mg THC/2.5 mg CBD per day to reach 7.5 mg THC/7.5 mg CBD per day until the end of treatment. (**B**) CBD arm: 3 participants reached the maximum daily dose of 800 mg CBD after 4 weeks of treatment, but two of them experienced AEs (transient transaminitis for #102107) and SAE (hepatitis with persistent elevated transaminases and worsened diabetes type 2, for #102108) and the treatment was permanently discontinued 1 and 2 weeks after, and they were withdrawn from the study. The other participant who reached the maximum daily dose of 800 mg remained on this daily dose until the end of the study. Finally, two participants from arm 2 gradually increased their daily dosing to reach dose of 400 mg of CBD per day and remained in this range until the end of the study medication.

**Figure 4:**
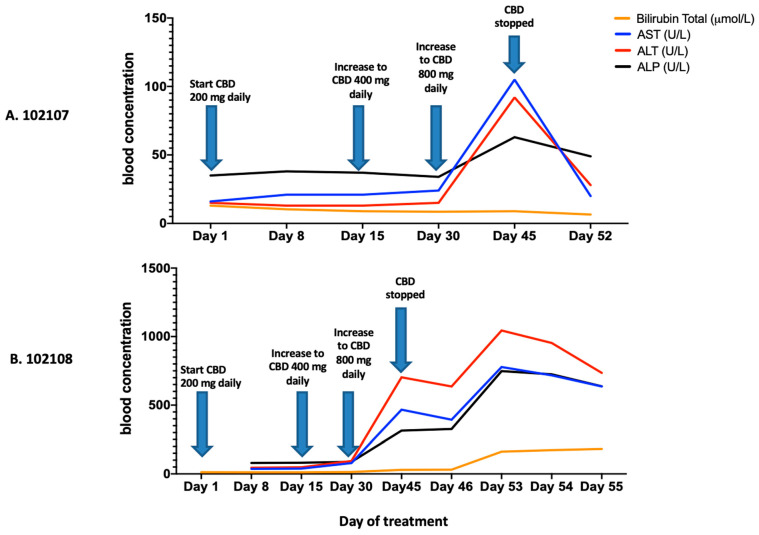
Dynamics of liver enzymes of participant #102107 and #102108 from arm 2 (TN-C200M2: CBD only), during cannabinoid uptake. Evolution of liver enzyme blood levels in (**A**). participant #102107 and (**B**). participant #102108 during the up-titration of CBD dose from the starting of CBD medication to the cessation of the treatment.

Dosing completion and Adverse Events (AE): Dosing based on participant self-titration is depicted in Figure 3. The majority of participants experienced AEs that were mild or moderate in severity. Eight out of 10 participants completed 12 weeks of treatment. Two participants were withdrawn from the CBD-only arm at 6 weeks for safety concerns. In one case, the 33-year-old female had grade 2 anemia (Hg 83 g/L) at screening, which failed to improve with oral iron supplementation. With frequent phlebotomy, the anemia progressed to grade 3 (lowest Hg 76 g/L) over the course of the study. She was withdrawn to prevent aggravation of her anemia. She also had moderate transaminitis at week 6 which normalized within 1 week following CBD cessation (Figure 4). The second participant withdrawn was a 62-year-old male who developed acute hepatitis at 6 weeks (Figure 4), deemed to be an unexpected, life-threatening serious adverse events (SAE), possibly related to cannabinoid treatment and requiring permanent CBD discontinuation and hepatology evaluation. Imaging revealed a pancreatic head mass, confirmed by pathology as pancreatic adenocarcinoma. Other potential contributors rendering the participant at risk for hepatitis included diabetes type 2, mild hepatic steatosis (diagnosed by abdominal ultrasound), alcohol binging, and possible interaction with ART resulting in increased CBD levels. Due to these two episodes of hepatitis, the protocol was amended and the maximum allowed dose for the CBD-only arm was reduced to 400 mg daily. However, this new protocol amendment only affected one individual.

Eight out the 10 study participants (80%) reported at least one AE, including 4 out of 5 individuals in each arm. The most commonly reported AE was somnolence (50%), followed by diarrhea (20%), difficulty concentrating (20%), transaminitis (20%) and worsened diabetes type 2 (20%). Only somnolence, difficulty concentrating, cognitive impairment and increase appetite were considered definitively related to cannabinoids and resolved with dose reduction. Apart from the SAE, the majority of AEs were of mild-moderate severity. Other AEs, as listed in Table 3, were reported only once and were considered possibly, probably or not related to treatment (10%).

### 3.3. Hematology, Biochemistry, and HIV Immunology and Virology

As previously indicated, one participant (CBD-only arm) who started the study with a known anemia (83 G/L) of grade 2, progressed to anemia of grade 3 at visit 5 and 6 (Supplementary Table S3). Two diabetic participants developed worsened glucose control. One participant, from the THC/CBD arm, had a glucose which progressively reached, at the end of treatment (Visit 9), a WHO toxicity grade 3 (Supplementary Table S4). The other diabetic was the participant who experienced the SAE. His ALT rise progressed to WHO toxicity grade 4 by Week 6. His blood glucose was of WHO toxicity grade 3 at Weeks 4 and 6 (Figure 4 and Supplemental Table S4). Apart from these diabetic participants, we did not observe any significant changes in glucose control (Table 4). Moreover, cannabinoids did not affect CD4 and CD8 count, nor HIV viral load (Supplementary Table S5). Therefore, in the overall study, 7 out of 10 individuals (70%) did not experience any significant toxicity (Grades 0–2 scores on the WHO toxicity scale).

### 3.4. Quality of Life and Mood Assessment

EQ-5D. Supplementary Figure S1 depicts the distribution of responses to the EQ-5D questionnaire at Baseline 2 (prior to treatment), Visit 6 (during treatment) and Visit 9 (end of treatment). Overall, cannabinoids did not significantly affect QOL.

WHOQOL-HIV BREF. Similar to what was observed with the EQ-5D questionnaire, at baseline and throughout cannabinoid treatment, the majority of participants reported moderate to very good quality of life (100%) and general health (80%, 90% and 100%, at Baseline 2, Visit 6 and Visit 9, respectively) (Supplementary Figure S2). Variations in scores across visits were not significant (Supplementary Figure S2).

POMS. Five out of ten participants (50%) showed a reduction in total mood disturbance (TMD) score over time. Three participants showed a slight increase in their TMD over time, including one in the THC/CBD arm, and 2 in the CBD-only arm who were withdrawn from the study. Variations in scores across visits were not significant (Supplementary Figure S3).

## 4. Discussion

In this pilot clinical trial, we evaluated the safety and tolerability of oral THC:CBD combination and CBD-only capsules over a 12 week period in PLWH on effective ART. Overall, the capsules were safe and well-tolerated, with AEs mostly mild to moderate in severity. Most participants completed the full 12 weeks of treatment. Importantly, even at the highest dose, capsules did not negatively affect immunological (CD4 and CD8 T cells counts) or virological (HIV viral load) parameters associated with HIV infection.

Highly purified THC:CBD combination and CBD-only capsules demonstrated acceptable safety and tolerability profiles in clinical trials for persons with epilepsy, chronic pain, and symptoms associated with multiple sclerosis and cancers [40,45,46,47,48,49,50,51,52,53,54,55,56]. In these trials, the common cannabinoid-related AEs were mostly somnolence, diarrhea, abdominal pain, fatigue, nausea, dry mouth, or dizziness, which occurred mainly during the up-titration and were considered mild or moderate in severity and resolved without discontinuation of the cannabinoid [40,45,46,47,48,49,50,51,52,53,54,55,56]. Similar to these studies, our participants also experienced somnolence and diarrhea as common AEs regardless of study arm. Abdominal pain, fatigue, nausea, dry mouth or dizziness were also reported, but less frequently. All these AEs were mild or moderate in severity and none resulted in treatment discontinuation.

A key observation from this study is the potential risk for transaminitis and hepatotoxicity, particularly for high doses of CBD (800 mg per day) in PLWH. In our study, two participants experienced an abnormal rise in their ALT serum levels above the upper limit of the normal range during the up-titration of CBD from dose 400 to 800 mg. Transient CBD-related elevations of ALT and AST are commonly reported in clinical studies [40,46,47,48,50,54,56]. Transient transaminase abnormalities do not seem to be of critical long-term clinical significance for the liver as they tend to normalize following dose reduction or treatment discontinuation [57,58]. On the other hand, the second participant who experienced abnormally high elevated transaminases was a 62 years old white man with underling fatty liver disease with elevated levels of transaminases (ALT and AST) and blood glucose before the initiation of CBD treatment, along with alcohol binging which was not openly disclosed to the study team. This participant presented a significant and persistent rise of ALT, AST, ALP and total bilirubin, with an increase in blood glucose levels. In that case, the persistency of the transaminitis, even after treatment discontinuation, prompted us to conduct further liver examinations which revealed a pancreatic adenocarcinoma. The underlying pathological state of this participant with several serious comorbidities and pre-existing elevated transaminases would have facilitated the marked rise of transaminases during the up-titration as shown in another clinical trial where participants having elevated baseline serum ALT had 3 fold greater incidence of significant ALT elevations compared to those starting CBD with normal level of ALT [56]. The likely involvement of high doses of CBD in the aggravation of the pathological state of the participant cannot be excluded [56]. Together, these findings suggest that HIV physicians should consider screening PLWH with risk factors for hepatic steatosis with transient elastography (Fibroscan^®^, Echosens, Paris, France) before initiating cannabinoid-based medicines given the high number of baseline comorbidities and risks for chronic liver disease in this specific population [59]. Study teams should follow liver enzymes closely to detect any subtle rises in transaminases which may suggest an undiagnosed steatohepatitis. Following the SAE in the later patient, we reduced the maximum dose of CBD to 400 mg po daily in the CBD-only arm. As CBD oils are available for purchase without prescription in Canada and some other jurisdictions, PLWH who use these products should be counselled about their potential hepatotoxicity.

Although two participants in our study had worsened blood glucose control, in one case this was the same participant with the SAE, suggesting that this could have been induced by binge drinking. In the other participant, although less probable, a potential drug interaction between cannabinoid treatment and one of the other medications taken by the participant could not be entirely excluded. Cannabinoids can alter hepatic metabolism of other drugs, making them ineffective or toxic [60,61]. The roles of cannabinoids in glucose metabolism and diabetes have been documented, though mostly in in vitro and animal studies, and suggest beneficial, rather than deleterious effects on diabetic parameters [62,63,64,65,66,67,68,69]. Similarly, observational studies in individuals using cannabis more often suggest a protective effect of cannabis use against metabolic syndrome and diabetes mellitus [70,71,72]. In another pilot clinical trial assessing the effect of cannabinoids on glycemic parameters in diabetic individuals, although CBD failed to directly improve diabetes parameters, it did not worsen glucose levels [73]. The findings from these other studies suggest that the uncontrolled blood glucose levels observed in two participants in our trial are unlikely due to a direct effect of cannabinoids, but rather to a combination of factors including comorbidities, alcohol consumption and polypharmacy.

Improvement in quality of life and mood are primary reasons why many PLWH use cannabis [74], although cannabinoids did not impact on quality of life or on mood scores throughout the study. However, most participants had good quality of life and mood scores at baseline, perhaps making it difficult to observe significant improvements. Future studies may wish to enroll individuals with poor or moderate mood or quality of life scores at baseline in order to appreciate whether any improvement occurs with treatment. Other studies have shown that cannabis in PLWH [12] or individuals with chronic pain [59] was associated with a marked improvement in quality of life [12,59].

## 5. Study Limitations

The most important limitation of this pilot study is the small sample size, greatly limiting our ability to generalize these findings to other PLWH. We encountered significant difficulty recruiting participants for this study. The lack of a placebo group and predominance of male participants are other limitations. While THC:CBD capsules were overall well-tolerated in the 5 participants who received them, there was likely a self-selection bias, with individuals participating in the study being more open towards the therapeutic potential of cannabinoids and also more experienced with cannabinoids than those who declined participation.

## 6. Conclusions

Taken together, our findings suggest that much additional work is needed to understand safety and tolerability in PLWH. Transaminitis in 2/5 participants in the CBD-only group suggests that more work is required to elucidate the best dosages of CBD in this population. Pharmacokinetic studies in this regard could be helpful. We suggest that PLWH should undergo a hepatological screening, ideally with transient elastography or, if unavailable, with simple fibrosis biomarkers like fibrosis-4 score, prior to initiating cannabinoid-based medicine, particularly formulations with high CBD dose, and they should be closely monitored to detect any rise of transaminases reflecting potential hepatotoxicity. Work is also required to better understand the potential benefits and harms of cannabinoids in chronic liver diseases and particularly in the context of HIV. As we recently reviewed [69], the liver contains both CB1 and CB2 receptors and the consequences of administering compounds which target these receptors must be understood. While future studies in PLWH should use a lower maximum dose of CBD, the optimal dose to avoid hepatotoxicity while leveraging anti-inflammatory and immunomodulatory properties of cannabinoids are unknown. Future studies may wish to examine the potential of these compounds to improve specific conditions, such as fatty liver disease, in PLWH. Work is currently ongoing to address whether the treatments in this study had any impact on immune activation, inflammatory markers, HIV reservoir size or gut microbiome, the ultimately goal being a reduction in HIV-associated comorbidities driven by chronic inflammation. Given the small sample size and lack of blinding or use of placebo, we remain cautious in our conclusions regarding the therapeutic benefits until more data is available.

## Figures and Tables

**Table 1:** Recommended up-titration schedule for TN-TC11M2 and TN-C200M2 regimens.

Arm 1 (TN-TC11M2: (THC: 2.5 mg/CBD: 2.5 mg))			Arm 2 (TN-C200M2: CBD; 200 mg) Original Titration Schedule *			Arm 2 (TN-C200M2: CBD; 200 mg) Revised Titration Schedule ^#^		
Weeks	Daily Dose	Number of Capsules (Taken Orally and Spaced Out Every 12 h)	Weeks	Daily Dose	Number of Capsules (Taken Orally and Spaced Out Every 12 h)	Weeks	Daily Dose	Number of Capsules (Taken Orally and Spaced Out Every 12 h)
Week 0 and 1 (Day 1–14)	5 mg THC/5 mg CBD	1 capsule twice daily (2 capsules per day)	Week 0 and 1 (Day 1–14)	200 mg CBD	1 capsule once daily	Week 0 and 1 (Day 1–14)	200 mg CBD	1 capsule once daily
Week 2 and 3 (Day 15–28)	10 mg THC/10 mg CBD	2 capsules twice daily, (4 capsules per day)	Week 2 and 3 (Day 15–28)	400 mg CBD	1 capsule twice daily (2 capsules per day)	Week 2–11 (Day 15–84)	400 mg CBD	1 capsule twice daily (2 capsules per day)
Week 4–11(Day 29–84)	15 mg THC/15 mg CBD	2 capsules three times daily,(6 capsules per day)	Week 4–11(Day 29–84)	800 mg ^#^ CBD	2 capsules twice daily(4 capsules per day)			

* Original titration schedule for arm 2: this titration schedule has been revised because of possible hepatotoxicity of high dose of CBD (800 mg per day); ^#^ Revised titration schedule for arm 2.

**Table 2:** Demographic and biological characteristics of study participants at inclusion (*n* = 10).

	Total Population	THC:CBD Arm (*n* = 5)	CBD Arm (*n* = 5)
**Age (Years), median (** **±IQR)**	57.5 (54.75–61.75)	57.0 (46.5–57.5)	62 (47.0–65.0)
**Sex assigned at birth (*n* (%))**			
Male	8 (80%)	5 (100%)	3 (60%)
Female	2 (20%)	0 (0%)	2 (40%)
**Ethnicity (*n* (%))**			
White-North American	6 (60%)	3 (60%)	3 (60%)
Black-African	1 (10%)	0 (0%)	1 (20%)
Asian	1 (10%)	1 (20%)	0 (0%)
Mixed ethnicity	2 (20%)	2 (40%)	0 (0%)
**Marital status (*n* (%))**			
Single	5 (50%)	2 (40%)	3 (60%)
Living as married	3 (30%)	2 (40%)	1 (20%)
Married	0 (0%)	0 (0%)	0 (0%)
Divorced	2 (20%)	2 (40%)	0 (0%)
Widowed	0 (0%)	0 (0%)	0 (0%)
**Highest education level (*n* (%))**			
Elementary (grade) school	0 (0%)	0 (0%)	0 (0%)
Secondary (High) school diploma	3 (30%)	1 (20%)	2 (40%)
College diploma	2 (20%)	1 (20%)	1 (20%)
Apprenticeship or trades certificate or diploma	1 (10%)	1 (20%)	0 (0%)
Bachelor’s degree	2 (20%)	1 (20%)	1 (20%)
Professional degree (e.g., MD, PharmD)	0 (0%)	0 (0%)	0 (0%)
Graduate degree (Master or Doctorate)	2 (20%)	2 (40%)	0 (0%)
**Cannabis use in the past 6 months (*n* (%))**			
No	3 (30%)	2 (40%)	1 (20%)
Yes	7 (70%)	3 (60%)	4 (80%)
Monthly	5 (72.43%)	2 (40%)	3 (60%)
Weekly	2 (28.57%)	1 (20%)	1 (20%)
Daily	0 (0%)	0 (0%)	0 (0%)
**Alcohol use in the past 6 months (*n* (%))**			
No	5 (50%)	2 (40%)	3 (60%)
Yes	5 (50%)	3 (60%)	2 (40%)
**Drug use in the past 6 months (*n* (%))**			
No	3 (30%)	2 (40%)	1 (20%)
Yes	7 (70%)	3 (60%)	4 (80%)
**History of infectious diseases (*n* (%))**			
Syphilis (treated)	2 (20%)	2 (40%)	0 (0%)
Hepatitis B (Anti-Hepatitis B core antibodies)	4 (40%)	2 (40%)	2 (40%)
Hepatitis C (Anti-Hepatitis C Antibodies)	0 (0%)	0 (0%)	0 (0%)

**Table 3:** List of adverse events (AEs) experienced by the participants during the study.

Adverse Events	Total Population (*n* = 10) (*n* (%))	THC:CBD Arm (*n* = 5) (*n* (%))	CBD Arm (*n* = 5) (*n* (%))
Somnolence	5 (50%)	2 (40%)	3 (60)
Diarrhea	2 (20%)	1 (20%)	1 (20%)
Difficulty concentrating	2 (20%)	1 (20%)	1 (20%)
Transaminitis	2 (20%)	0 (0%)	2 (40%)
Worsened diabetes type 2	2 (20%)	1 (20%)	1 (20%)
Abdominal cramps	1 (10%)	1 (20%)	0 (0%)
Acute hepatitis *	1 (10%)	0 (0%)	1 (20%)
Altered perception of peripheral neuropathy of feet bilaterally	1 (10%)	1 (20%)	0 (0%)
Bilateral leg weakness	1 (10%)	0 (0%)	1 (20%)
Cognitive impairment	1 (10%)	1 (20%)	0 (0%)
Constipation	1 (10%)	0 (0%)	1 (20%)
Dental abscess	1 (10%)	1 (20%)	0 (0%)
Dizziness	1 (10%)	0 (0%)	1 (20%)
Dry mouth	1 (10%)	1 (20%)	0 (0%)
Fatigue	1 (10%)	1 (20%)	0 (0%)
Gastroenteritis	1 (10%)	0 (0%)	1 (20%)
Hypocalcemia	1 (10%)	0 (0%)	1 (20%)
Increase appetite	1 (10%)	1 (20%)	0 (0%)
Nausea	1 (10%)	1 (20%)	0 (0%)
Nightmares	1 (10%)	1 (20%)	0 (0%)
Palpitations	1 (10%)	0 (0%)	1 (20%)
Paranoid thoughts	1 (10%)	1 (20%)	0 (0%)
Right sided cramps	1 (10%)	0 (0%)	1 (20%)
Upper tract respiratory infection	1 (10%)	0 (0%)	1 (20%)
Worsened anemia	1 (10%)	0 (0%)	1 (20%)
Worsened renal function	1 (10%)	0 (0%)	1 (20%)

* Life threatening, serious adverse event.

**Table 4:** Liver enzyme and kidney function profiles.

(Median (IQR))	Screening Visit	Visit 3	Visit 4	Visit 5	Visit 6	Visit 7	Visit 8	Visit 9	*p*-Value Friedman Test	*p*-Value Wilcoxon Matched-Pairs Signed Rank Test (Visit 3 vs. Visit 9)
Creatinine (μmol/L) ^$^	86.0 (70.5–100.0)	87.0 (68.75–105.3)	88.50 (66.50–103.5)	89.00 (71.75–104.5)	92.00 (69.75–104.5)	90.0 (77.0–104.8)	102.0 (76.5–109.5)	80.0 (66.5–103.0)	0.49	0.55
Alanine Aminotransferase (ALT) (U/L) ^€^	19.5 (13.75–37.0)	21.5 (11.5–36.75)	19.5 (12.0–41.25)	19.0 (13.25–34.0)	21.0 (14.25–50.75)	20.5 (9.5–31.25)	20.5 (11.0–25.5)	23.0 (14.0–35.5)	0.31	0.87
Aspartate Aminotransferase (AST) (U/L)	19.0 (13.5–28.0)	18.5 (15.75–26.5)	20.5 (14.75–30.0)	17.0 (14.5–23.5)	19.5 (15.5–48.75)	18.0 (12.0–22.75)	18.0 (10.0–21.0)	20.0 (15.0–28.5)	0.76	>0.99
Alkaline Phosphatase (ALP) (U/L)	68.0 (56.25–83.5)	65.0 (55.5–73.25)	61.0 (52.5–70.5)	58.5 (53.25–78.5)	63.5 (54.75–77.5)	60.0 (51.5–72.5)	60.0 (55.0–62.0)	61.0 (54.0–73.5)	0.0107 *	0.29
Urea (mmol/L) ^£^	6.1 (4.4–7.43)	5.9 (3.87–7.45)	6.3 (4.55–6.83)	6.25 (4.33–7.13)	6.15 (4.95–7.85)	5.95 (4.45–7.67)	6.1 (4.5–8.63)	6.0 (4.4–7.05)	0.38	0.71
Blood Glucose (mmol/L)	5.2 (4.87–6.97)	5.5 (4.57–7.3)	5.3 (4.9–9.4)	5.6 (4.93–9.77)	5.7 (5.1–9.1)	5.0 (4.6–6.13)	5.2 (4.95–5.37)	5.1 (5.0–6.15)	0.38	0.46
Total Bilirubin (μmol/L)	10.15 (7.65–12.25)	9.4 (7.87–11.33)	10.0 (7.65–11.53)	8.8 (6.8–15.05)	9.0 (7.67–11.40)	8.05 (7.1–9.57)	9.3 (7.65–10.0)	8.5 (7.05–12.20)	0.98	0.64

^$^ μmol/L: Micromolar per liter; ^€^ U/L: Units per liter; * while statistically significant, this was not deemed to be clinically significant. ^£^ mmol/L: Millimolar per liter.

## Data Availability

Anonymized data may be available upon reasonable request of the author and the CTN.

## Supplementary Materials

The following supporting information can be downloaded at: https://www.mdpi.com/article/10.3390/biomedicines10123168/s1, Figure S1. Frequency of responses of the EuroQoL-5D (EQ-5D) questionnaire for the quality-of-life assessment during the 12 weeks of treatment. Figure S2. Frequency of responses of the World Health Organization Quality of Life—HIV Brief (WHOQOL-HIV BREF) questionnaire for general quality of life and general health state during the study visits. Figure S3. Total mood disturbance (TMD) score from the Profile of Mood States) (POMS) questionnaire during the study. Table S1. List of exclusion criteria. Table S2. World Health Organization (WHO) Toxicity Scale Grading for Adverse Events. Table S3. Hematological parameters (excel attachment). Table S4. Biochemistry parameters (excel attachment). Table S5. HIV immunological and virological parameters.
