# Supplementary material for: Safety and Tolerability of Oral Cannabinoids in People Living with HIV on Long-Term ART: A Randomized, Open-Label, Interventional Pilot Clinical Trial (CTNPT 028)
Source: Biomedicines. 2022 Dec 7;10(12):3168. doi: 10.3390/biomedicines10123168 (PMC9775551; doi:10.3390/biomedicines10123168)
Supplement: Supplementary file 1 [file biomedicines-10-03168-s001.zip › biomedicines-2033904-supplementary.pdf]

Supplementary Figures

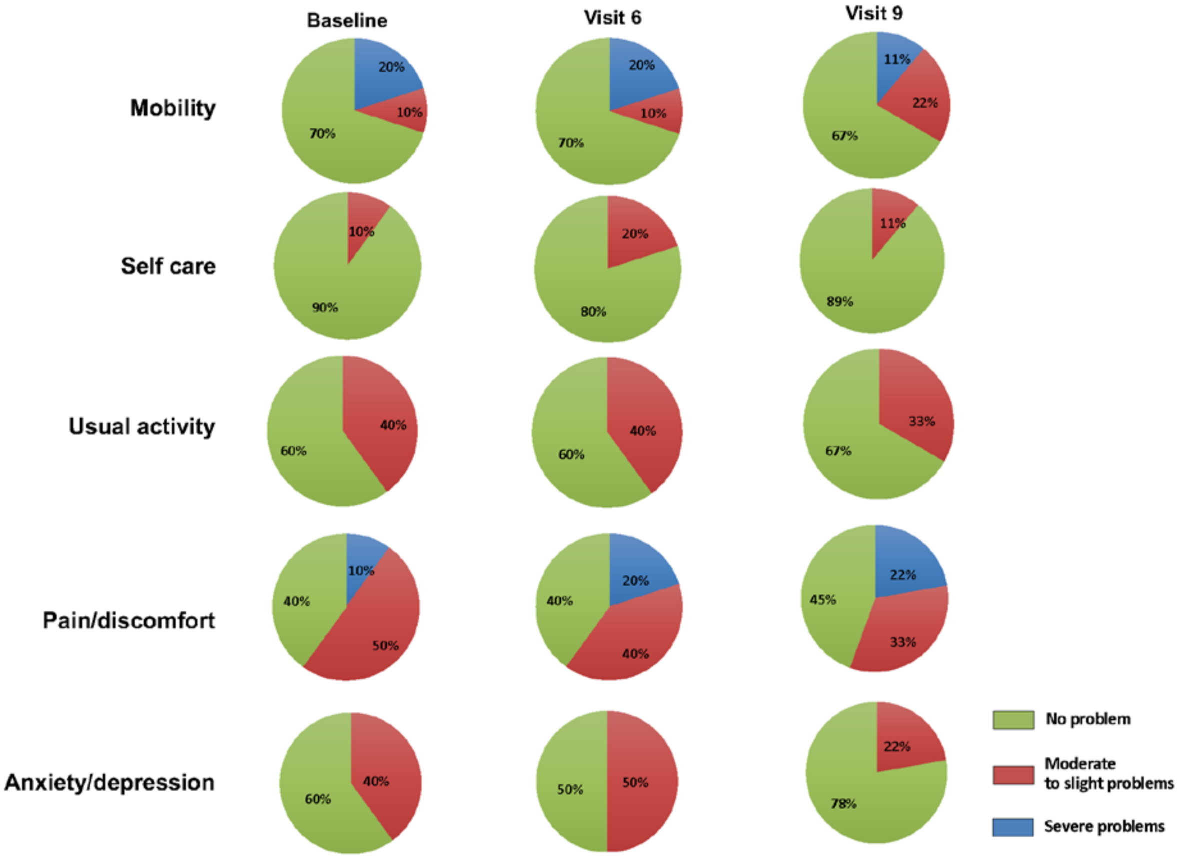

Supplementary Figure S1. Frequency of responses of the EQ-5D questionnaire for the quality-of-life assessment during the 12 weeks of treatment. Frequency of the different responses possible, "no problem", "slight to moderate problem" and "severe problem", for the 5 domains (Anxiety/depression; pain/discomfort; usual activity; self-care; and mobility) of the EQ-ED questionnaires.

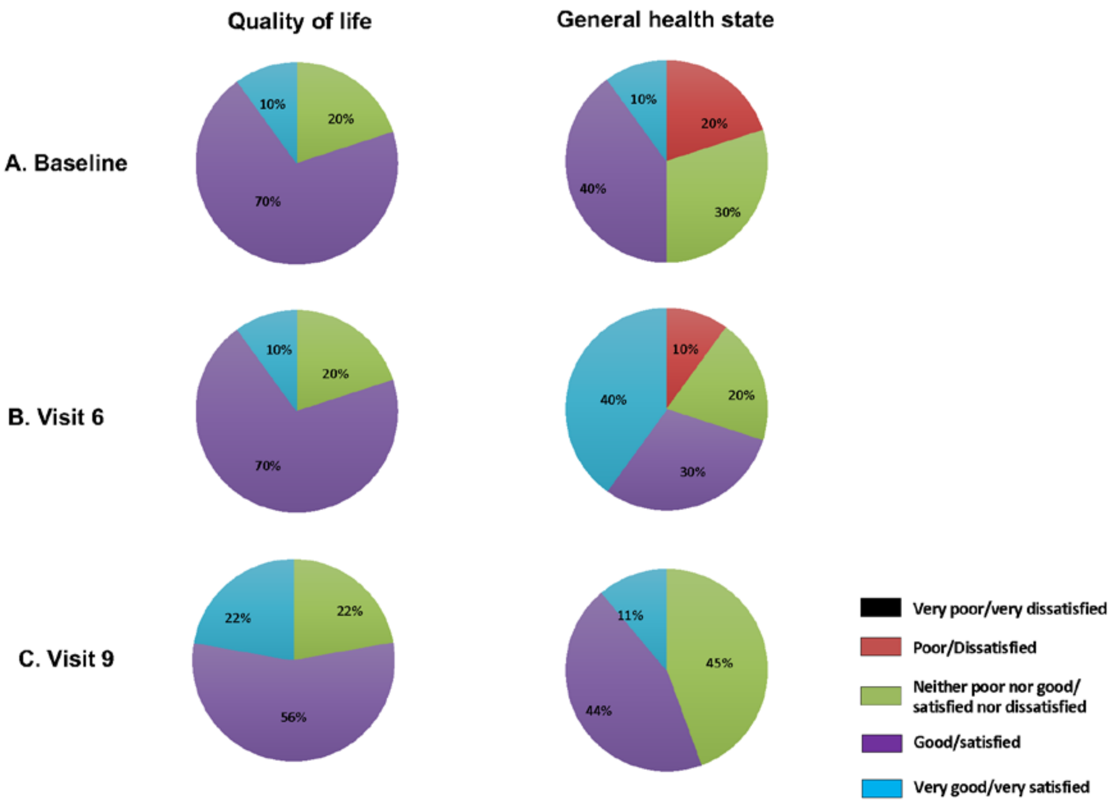

Supplementary Figure S2. Frequency of responses of the WHOQOL-HIV BREF questionnaire for general quality of life and general health state during the study visits.

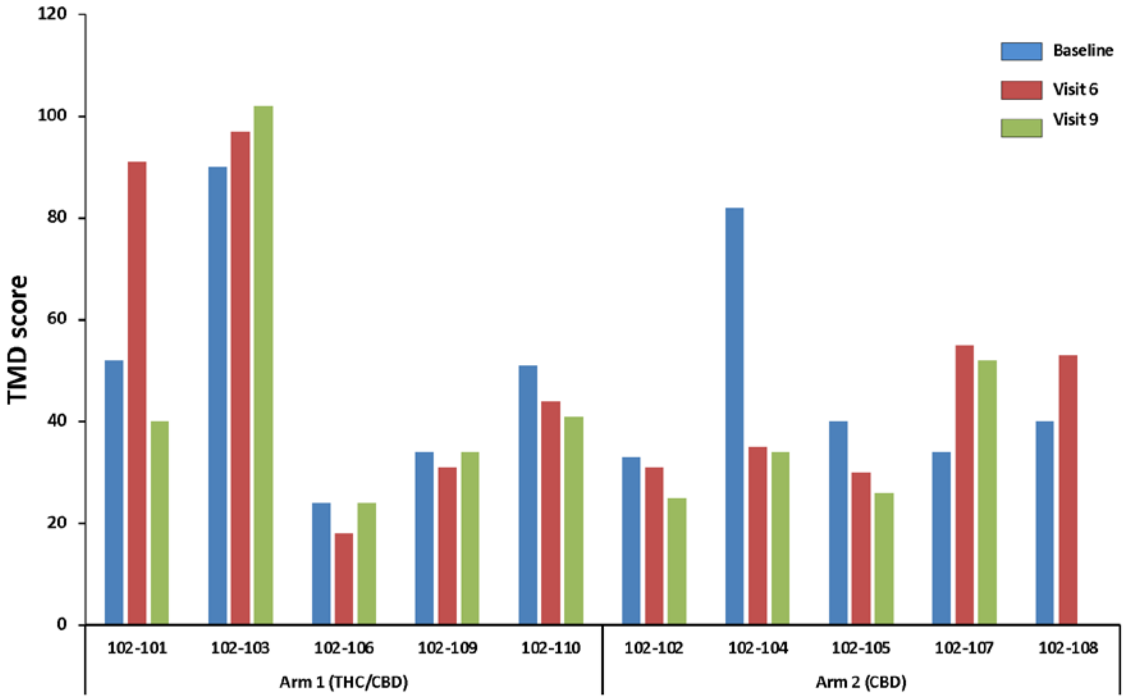

Supplementary Figure S3. Total mood disturbance (TMD) score from the POM questionnaire during the study

Supplemental Table S1. List of exclusion criteria

List of exclusion criteria
• Using cannabinoid-containing products outside of the study or within 4 weeks of study commencement;<br>• Being pregnant, breast feeding or planning to become pregnant during the course of the study;<br>• Being enrolled in a separate study involving administration of medication, vitamin, supplement or herbal product;<br>• Being active intravenous drug user or active substance dependent;<br>• Having a prior history of hypersensitivity to cannabis or cannabis-containing products;<br>• Having a known or suspected allergy to peppermint oil;<br>• Having a diagnosed active opportunistic infection or malignant condition;<br>• Having unintentional weight loss of 10% or more of body weight in the last 6 months, having experienced an unstable angina or acute cardiac event in the past year;<br>• Having active psychiatric disorders or history of psychiatric depression (other than mild depression or anxiety), being on antipsychotic medication;<br>• Having known or suspected family history of schizophrenia or severe personality disorder;<br>• Having serious cardiovascular disease such as ischemic disease, arrhythmias, poorly controlled hypertension or severe heart failure, experiencing anemia (hemoglobin <100 g/L), active liver disease or unexplained persistent elevations of serum transaminases;<br>• Being co-infected with hepatitis B or C (positive Hepatitis B Surface antigen (HBsAg) or positive anti-HBc (Hepatitis B Core) antibodies with a detectable Hepatitis B virus (HBV) DNA viral load or positive anti-HCV antibodies with a detectable Hepatitis C virus (HCV) RNA viral load);<br>• Having alanine aminotransferase (ALT) or aspartate aminotransferase (AST) or alkaline phosphatase (ALP) >2.5 × upper limit of normal, suffering from renal dysfunction;<br>• Holding employment that requires operation of heavy machinery or which requires undergoing drug screening (ie, pilot or police officer);<br>• Concurrent use within the past 8 weeks of anabolic hormones, prednisone, IL-2 or other agents known to alter immune function

Note: Non-alcoholic fatty liver disease (NAFLD) was not considered active liver disease if LFTs were normal

Supplementary Table S2. WHO Toxicity Scale Grading for Adverse Events.

<u>ABBREVIATIONS</u>

Abbreviations used in the Table:
ULN=Upper Limit of Normal
LLN=Lower Limit of Normal
Rx=Therapy
Req=Required
Mod=Moderate
IV=Intravenous
ADL=Activities of Daily Living
Dec=Decreased

<u>ESTIMATING SEVERITY GRADE</u>

For abnormalities NOT found elsewhere in the Toxicity Tables use the scale below to estimate the grade of severity:

GRADE 1: Mild, transient or mild discomfort (<48 hours); no medical intervention/therapy required
GRADE 2: Moderate, mild to moderate limitation in activity—some assistance may be needed; no or minimal medical intervention/therapy required
GRADE 3: Severe, marked limitation in activity, some assistance usually required; medical intervention/therapy required, hospitalizations possible
GRADE 4: Life threatening, extreme limitation in activity, significant assistance required; significant medical intervention/therapy
required, hospitalization or hospice care probably

(a) Hematology

	Grade 1	Grade 2	Grade 3	Grade 4
Hemoglobin	9.5-10.5 g/dL	8.0-9.4 g/dL	6.5-7.9 g/dL	<6.5 g/dL
Absolute Neutrophil Count	1000-1500/mm^3	750-999/mm^3	500-749/mm^3	<500 mm^3
Platelets	75,000-99,999/mm^3	50,000-74,999/mm^3	20,000-49,999/mm^3	<20,000 mm^3
WBCs	11,000-13,000/mm^3	13,000-15,000/mm^3	15,000-30,000/mm^3	>30,000-<1,000/ mm^3
% Polymorphonuclear Leukocytes + Band Cells	>80%	90-95%	>95%	---
Abnormal Fibrinogen	Low: 100-200 mg/dL	Low: <100 mg/dL	Low: <50 mg/dL	Fibrinogen associated with

	High: 400-600 mg/dL	High: >600 mg/dL	----	gross bleeding or with disseminated intravascular coagulation
Fibrin Split Product	20-40 mcg/mL	41-50 mcg/mL	51-60 mcg/mL	>60 mcg/mL
Prothrombin Time (PT)	1.01-1.25 x ULN	1.26-1.5 x ULN	1.5-3.0 x ULN	>3 x ULN
Activated Partial Thromboplastin (APPT)	1.01-1.66 x ULN	1.67-2.33 x ULN	2.34-3 x ULN	> 3 x ULN
Methemoglobin	5.0-9.9%	10.0-14.9%	15.0-19.9%	>20.0%

(b) CHEMISTRIES

	Grade 1	Grade 2	Grade 3	Grade 4
Hyponatremia	130-135 mEq/L	123-129 mEq/L	116-122 mEq/L	<116 mEg/L or abnormal sodium with mental status change or seizures
Hypernatremia	146-150 mEq/L	151-157 mEq/L	158-165 mEq/L	>165 mEq/L or abnormal sodium with mental status changes or seizures
Hypokalemia	3.0-3.4 mEq/L	2.5-2.9 mEq/L	2.2-2.4 mEq/L or intensive replacement therapy or hospitalization required	<2.0 mEq/L or abnormal potassium with paresis, ileus or life-threatening arrhythmia
Hyperkalemia	5.6-6.0 mEq/L	6.1-6.5 mEq/L	6.6-7.0 mEq/L	>7.0 mEq/L or abnormal potassium with life-threatening arrhythmia
Hypoglycemia	55-64 mg/dL	40-54 mg/dL	30-39 mg/dL	<30 mg/dL or abnormal glucose with mental status changes or coma

Hyperglycemia (nonfasting and no prior diabetes)	116-160 mg/dL	161-250 mg/dL	251-500 mg/dL	>500 mg/dL or abnormal glucose with ketoacidosis or seizures
Hypocalcemia (corrected for albumin)	8.4-7.8 mg/dL	7.7-7.0mg/dL	6.9-6.1 mg/dL	<6.1 mg/dL or abnormal calcium with life-threatening arrhythmia or tetany
Hypercalcemia (corrected for albumin)	10.6-11.5 mg/dL	11.6-12.5 mg/dL	12.6-13.5 mg/dL	>13.5 mg/dL or abnormal calcium with life threatening arrhythmia
Hypomagnesemia	1.4-1.2 mEq/L	1.1-0.9 mEq/L	0.8-0.6 mEq/L	<0.6 mEq/L or abnormal magnesium with life-threatening arrythmia
Hypophosphatemia	2.0-2.4 mg/dL	1.5-1.9 mg/dL or replacement Rx required	1.0-1.4 mg/dL intensive therapy or hospitalization required	<1.0 mg/dL or abnormal phosphate with life-threatening arrhythmia
Hyperbilirubinemia	1.1-1.5 x ULN	1.6-2.5 x ULN	2.6-5 x ULN	> 5 x ULN
BUN	1.25-2.5 x ULN	2.6-5 x ULN	5.1-10 x ULN	>10 x ULN
Hyperuricemia (uric acid)	7.5-10.0 mg/dL	10.1-12.0 mg/dL	12.1-15.0 mg/dL	>15.0 mg/dL
Creatinine	1.1-1.5 x ULN	1.6-3.0 x ULN	3.1-6 x ULN	>6 x ULN or dialysis required

(c) ENZYMES

	Grade 1	Grade 2	Grade 3	Grade 4
AST (SGOT)	1.25-2.5 x ULN	2.6-5 x ULN	5.1-10 x ULN	>10 x ULN
ALT (SGPT)	1.25-2.5 x ULN	2.6-5 x ULN	5.1-10 x ULN	>10 x ULN
GGT	1.25-2.5 x ULN	2.6-5 x ULN	5.1-10 x ULN	>10 x ULN
Alkaline Phosphatase	1.25-2.5 x ULN	2.6-5 x ULN	5.1-10 x ULN	>10 x ULN
Amylase	1.1-1.5 x ULN	1.6-2.0 x ULN	2.1-5.0 x ULN	>5.1 x ULN
Lipase	1.1-1.5 x ULN	1.6-2.0 x ULN	2.1-5.0 x ULN	>5.1 x ULN

(d) URINALYSIS

	Grade 1	Grade 2	Grade 3	Grade 4
Proteinuria	1+ or 200 mg-1g loss/day	2-3+ or 1-2 g loss/day	4+ or 2-3.5 g loss/day	Nephrotic syndrome or >3.5 g loss/day
Hematuria	Microscopic only <10 rbc/hpf	Gross, no clots >10 rbc/hpf	Gross, with or without clots, OR red blood cell casts	obstructive or required transfusion

(e) CARDIOVASCULAR

	Grade 1	Grade 2	Grade 3	Grade 4
Cardiac Rhythm		Asymptomatic, transient signs, no Rx required	Recurrent/persistent; symptomatic; Rx required	Unstable dysrhythmia; hospitalization and treatment required
Hypertension	Transient increase >20 mg/Hg; no treatment	Recurrent, chronic increase >20 mg/Hg/treatment required	Acute treatment required; outpatient treatment or hospitalization possible	End organ damage or hospitalization required
Hypotension	Transient orthostatic hypotension with heart rate increased by <20 beat/min or decreased by <10 mmHg systolic BP, no treatment required	Symptoms due to orthostatic hypotension or BP decreased by <20 mg Hg systolic; correctable with oral fluid treatment	Requires IV fluids; no hospitalization required	Mean arterial pressure <60 mg/Hg or end organ damage or shock; requires hospitalization and vasopressor treatment
Pericarditis	Minimal effusion	Mild/moderate asymptomatic effusion; no treatment	Symptomatic effusion; pain; EKG changes	Tamponade; pericardiocentesis or surgery required
Hemorrhage, Blood Loss	Microscopic/occult	Mild, no transfusion	Gross blood loss; 1-2 units transfused	Massive blood loss; >3 units transfused

(f) RESPIRATORY

	Grade 1	Grade 2	Grade 3	Grade 4
Cough	Transient; no treatment	Persistent cough; treatment responsive	Paroxysmal cough; uncontrolled with treatment	----
Bronchospasm, Acute	Transient; no treatment; FEV_1 70-80% of peak flow	Requires treatment; normalizes with bronchodilator; FEV_1 50-70% of peak flow	No normalization with bronchodilator; FEV_1 25-50% of peak flow; or retractions present	Cyanosis: FEV_1 <25% of peak flow or intubation necessary
Dyspnea	Dyspnea on exertion	Dyspnea with normal activity	Dyspnea at rest	Dyspnea requiring Oxygen therapy

Division of AIDS table for Grading Severity of Adult and Pediatric AEs, Dec. 2004:

Grade 1: Bronchospasm (acute) FEV1 or peak flow reduced to 70-80%

Grade 2: FEV1 or peak flow 50-69%

Grade 3: FEV1 or peak flow 25-49%

Grade 4: Cyanosis OR FEV1 or peak flow <25% OR Intubation

(g) GASTROINTESTINAL

	Grade 1	Grade 2	Grade 3	Grade 4
Nausea	Mild or transient; maintains reasonable intake	Moderate discomfort; intake decreased significantly; some activity limited	No significant intake; requires IV fluids	Hospitalization required
Vomiting	1 episode in 24 hours	2-5 episodes in 24 hours	>6 episodes in 24 hours or needing IV fluids	Physiologic consequences requiring hospitalization or requiring parenteral nutrition

	Grade 1	Grade 2	Grade 3	Grade 4
Constipation	Requiring stool softener or dietary modification	Requiring laxatives	Constipation requiring manual evacuation or enema	Obstruction or toxic megacolon
Diarrhea	Mild or transient; 3-4 loose stools/day or mild diarrhea last <1 week	Moderate or persistent; 5-7 loose stools/day or diarrhea lasting >1 week	>7 loose stools/day or blood diarrhea; or orthostatic hypotension or electrolyte imbalance or >2L IV fluids required	Hypotensive shock or physiologic consequences requiring hospitalization
Oral Discomfort/Dysphagia	Mild discomfort; no difficulty swallowing	Some limits on eating/drinking	Eating/talking very limited/ unable to swallow solid foods	Unable to drink fluids; requires IV fluids

(h) NEUROLOGICAL

	Grade 1	Grade 2	Grade 3	Grade 4
Neuro-cerebellar	Slight incoordination; dysdiadochokinesia	Intention tremor, dysmetria, slurred speech; nystagmus	Locomotor ataxia	Incapacited
Psychiatric	Mild anxiety or depression	Moderate anxiety or depression; therapy required; change in normal routine	Severe mood changes requiring therapy; or suicidal ideation; or aggressive ideation	Acute psychosis requiring hospitalization; or suicidal gesture/attempt or hallucinations
Muscle Strength	Subjective weakness; no objective symptoms/signs	Mild objective signs/symptoms	Objective weakness function limited	Paralysis

Paresthesia (burning, tingling, etc.)	Mild discomfort; no treatment required	Moderate discomfort; non-narcotic analgesia required	Severe discomfort; or narcotic analgesia required with symptomatic improvement	Incapacitating; or not responsive to narcotic analgesia
Neuro-sensory	Mild impairment in sensation (decreased sensation eg. Vibratory, pinprick, hot/cold in great toes) or focal area or symmetrical distribution; or change in taste, smell, vision and/or hearing	Moderate impairment (mod decreased sensation, eg. Vibratory, pinprick, hot/cold to ankles) and/or mild impairment that is not symmetrical	Severe impairment (decreased or loss of sensation to knees or wrists) or loss of sensation of at least mod degree in multiple different body areas (ie, upper and lower extremities)	Sensory loss involves limbs and trunk; paralysis; or seizures

(i) MUSCULOSKELETAL

	Grade 1	Grade 2	Grade 3	Grade 4
Arthralgia (joint pain)	Mild pain not interfering with function	Moderate pain, anagesics and/or pain interfering with function but not with activities of daily living	Severe pain; pain and/or analgesics interfering with activities of daily living	Disabling pain
Arthritis	Mild pain with inflammation, erythema or joint swelling—but not interfering with function	Moderate pain with inflammation, erythema or joint swelling—interfering with function, but not with activities of daily living	Severe pain with inflammation, erythema or joint swelling—and interfering with activities of daily living	Permanent and/or disabling joint destruction
Myalgia	Myalgia with no limitation of activity	Muscle tenderness (at other than	Severe muscle tenderness with marked	Frank myonecrosis

	Grade 1	Grade 2	Grade 3	Grade 4
		injection site) with moderate impairment of activity	impairment of activity	

(j) SKIN

	Grade 1	Grade 2	Grade 3	Grade 4
Mucocutaneous	Erythema; pruritis	Diffuse, maculopapular rash, dry desquamation	Vesiculation or moist desquamation or ulceration	Exfoliative dermatitis, mucous membrane involvement or erythema, multiforme or suspected Stevens-Johnson or necrosis requiring surgery
Induration	<15 mm	15-30 mm	>30 mm	
Erythema	<15 mm	15-30 mm	>30 mm	
Edema	<15 mm	15-30 mm	>30 mm	
Rash at Injection Site	<15 mm	15-30 mm	>30 mm	
Pruritis	Slight itching	Moderate itching at injection extremity	Itching over entire body	

(k) SYSTEMIC

	Grade 1	Grade 2	Grade 3	Grade 4
Allergic Reaction	Pruritis without rash	Localized urticaria	Generalized urticarial; angioedema	Anaphylaxis
Headache	Mild, no treatment required	Transient moderate; treatment required	Severe; responds to initial narcotic therapy	Intractable; requires repeated narcotic therapy
Fever: oral	37.7-38.5 C or 100.0-101.5F	38.6-39.5 C or 101.6-102.9F	39.6-40.5C or 103-105F	>40 C or >105F

Fatigue	Normal activity reduced <48 hours	Normal activity decreased 15-50% >48 hours	Normal activity decreased >50%; can't work	Unable to care for self

Supplementary Table S3. Hematological parameters (excel attachment)

Supplementary Table S4. **Biochemistry parameters** (excel attachment)

Supplementary Table S5. HIV immunological and virological parameters

**(Median (IQR))**	**Screening visit**	**Visit 4**	**Visit 6**	**Visit 9**	**P-value Friedman test**	**P-value Wilcoxon matched-pairs signed rank test (Screening vs visit 9)**
**CD4 count**	553.5 (472.3-674.5)	558.0 (477.5-714.0)	610.5 (498.3-807.5)	497.0 (383.0-645.5)	0.30	0.19
**CD8 count**	519.0 (460.5-842.5)	540.0 (414.8-765.3)	586.0 (421.5-896.8)	498.0 (381.0-780.0)	0.86	0.25
**CD4/CD8 ratio**	1.0 (1.0-1.0)	1.0 (1.0-1.0)	1.0 (1.0-1.0)	1.0 (0.95-1.0)	0.64	>0.99
**CD4 (%)**	37.0 (31.0-41.0)	36.5 (32.5-43.25)	37.0 (33.5-40.0)	36.0 (30.5-40.5)	0.09	0.34

**CD8 (%)**	35.0 (32.5-41.25)	35.0 (31.75-41.0)	34.0 (30.5-39.5)	37.0 (30.0-39.5)	0.57	0.62
**HIV RNA load**	Undetectable	Undetectable	Undetectable	Undetectable	NA*	NA

#Undetectable: below the lower limit of detection. *NA: Not applicable.
